# Dopamine Agonists and Pathologic Behaviors

**DOI:** 10.1155/2012/603631

**Published:** 2012-04-05

**Authors:** Brendan J. Kelley, Andrew P. Duker, Peter Chiu

**Affiliations:** Departments of Neurology and Psychology, University of Cincinnati, Suite 2300, Cincinnati, OH 45267, USA

## Abstract

The dopamine agonists ropinirole and pramipexole exhibit highly specific affinity for the cerebral dopamine D3 receptor. Use of these medications in Parkinson's disease has been complicated by the emergence of pathologic behavioral patterns such as hypersexuality, pathologic gambling, excessive hobbying, and other circumscribed obsessive-compulsive disorders of impulse control in people having no history of such disorders. These behavioral changes typically remit following discontinuation of the medication, further demonstrating a causal relationship. Expression of the D3 receptor is particularly rich within the limbic system, where it plays an important role in modulating the physiologic and emotional experience of novelty, reward, and risk assessment. Converging neuroanatomical, physiological, and behavioral science data suggest the high D3 affinity of these medications as the basis for these behavioral changes. These observations suggest the D3 receptor as a therapeutic target for obsessive-compulsive disorder and substance abuse, and improved understanding of D3 receptor function may aid drug design of future atypical antipsychotics.

## 1. Introduction

An association between neurodegeneration of the dopaminergic nigrostriatal system and the major motor symptoms of Parkinson's disease (PD) was first recognized in 1960 [1] after pioneering work by Arvid Carlsson showed that L-DOPA reversed the parkinsonian syndrome in rabbits induced by reserpine [2]. This observation led to the first trials of injected levodopa (L-dopa), a direct metabolic precursor of dopamine, to address motor symptoms associated with the disease. This treatment demonstrated transient success, but was impractical due to severe toxicities associated with the injections. Gradual titration of oral L-dopa was better tolerated, but was still associated with severe nausea and the requirement of higher doses of L-dopa due to peripheral consumption of the substrate. In the 1970s, compounding L-dopa with the peripheral dopa-decarboxylase inhibitor carbidopa very successfully addressed these shortcomings. Nausea and vomiting were reduced to such a degree that the medication adopted the trade name Sinemet (sine = without; emet = emesis). Compounded levodopa-carbidopa remains the mainstay of treatment for PD.

 Dopaminergic agonists are synthetic analogues of dopamine. Apomorphine was suggested for the treatment of PD as early as 1884 [3], although the first article describing its effectiveness was not published until 1951 [4]. Bromocriptine was found to be effective in PD in 1974 [5]. Other ergotamine dopamine agonists including lisuride, pergolide, and cabergoline were subsequently found to be effective. In the 1990s, two nonergot dopamine agonists (DA), pramipexole and ropinirole, were granted approval for use in the United States. These have been adopted by many clinicians for a variety of reasons, including a more stable motor response, improved side-effect profile, and more convenient dosing schedule.

As DA medicines became widely used, unanticipated reports of poorly modulated risk taking began to emerge, and the link between these behaviors and the medications was recognized by the year 2000 [6, 7]. These took the form of compulsive gambling, hypersexuality, hyperphagia, and even hobbying or shopping that took on an obsessive-compulsive-type character. Examining the pharmacology of these medications and their specificity to the D3 dopamine receptors provides an opportunity to understand why these pathological behaviors are not generally seen with levodopa, why tardive movement disorders arise in many patients taking typical (dopamine-targeting) neuroleptics, and why the recognition of DA-agonist-related pathological behaviors in PD patients may suggest potential therapeutic targets for similar behavioral problems that arise spontaneously in the general population.

## 2. Dopamine Receptors, L-Dopa, and Dopamine Agonists

Dopamine receptors have been divided into 5 different subtypes (D1–D5). Structurally, the D1 and D5 receptors are very similar, while the D2, D3, and D4 receptors are different from them. In particular, the D3 receptor has strong representation in the limbic system and its connections in the ventral striatum and is associated with cognitive, emotional, and endocrine functions [8].

L-dopa increases the availability of dopamine in the brain, without known specificity for a dopamine receptor subtype. In contrast, the dopamine agonists ropinirole, pramipexole, and pergolide exhibit high affinity for the D3 receptors [9–11]. The older dopamine agonist, bromocriptine, does not share this specificity and appears to have greater affinity for the D2 receptor [9].

This receptor specificity may have functional relevance to the increased rates of pathological behaviors, as the D3 receptor expression is particularly rich in limbic areas and often being coexpressed with D2 in regions serving sensory (sensory thalamic nuclei), hormonal (mammilothalamic tract), and association (amygdala) functions [12]. The D3 receptor appears to control the phasic, but not tonic, activity of dopaminergic neurons which may be induced by novelty or presentation of drug-conditioned cues in rodents [13–15]. These data seem to converge on an important role for the D3 receptor in modulating the physiologic and emotional experience of novelty, reward, and risk assessment and likely explain the relatively higher rates of pathological behaviors among patients taking DAs. Pathological behaviors associated with bromocriptine have not generally been observed, with a single case report in 2003 being the first time this association was noted [16]. This likely reflects the lower frequency of use and may also be understood in the context of bromocriptine lacking the D3 specificity of the more commonly utilized DAs. Animal models suggest that D3 receptor stimulation is also involved in the emergence of dopamine-induced dyskinesias [17, 18].

## 3. Pathological Behaviors

The most commonly reported pathological behaviors have been pathological gambling, hypersexuality, compulsive or binge eating, and compulsive shopping. Uncertainty remains regarding the overall frequency of DA-associated behavioral changes. Initial surveillance suggested very low rates—on the order of 2%–8% [19]. Subsequent structured-questionnaire ascertainments found higher rates, with a recent large questionnaire-based assessment reporting a rate of 13.6% [20]. This cross-sectional study assessed rates of pathologic gambling (9.9%), compulsive sexual behavior (4.4%), compulsive buying (7.2%), and binge eating (5.6%) among current DA users, with a total of 17.1% of current DA users exhibiting any pathological behavior. This compared to the significantly lower rate of pathological behaviors (6.9%) among subjects not using a DA for at least 6 months prior to enrollment.

Some authors argue that reliance on impersonal questionnaires or spontaneous patient reports likely results in incomplete ascertainment due to the sensitive and/or potentially embarrassing nature of these symptoms. Another recent report utilized physician-directed symptom elicitation and found pathological behaviors in 24% of patients using DA at therapeutic doses and in 30% of patients using “target” DA dosing [21]. Although involving a smaller population than some other reports, this paper highlights some difficulties in capturing behavioral changes with several patients exhibiting compulsive hobbying or computer use, and others having poor insight into their behavioral changes including a patient with compulsive gambling who perceived his behavior as “beneficial” due to net wins.

Emergence of pathological behaviors is very uncommonly seen among patients treated with L-dopa alone [22]. A large study utilizing structured interview assessment found pathological behaviors in 6.9% of subjects not currently taking a DA, although prior exposure to DA was not reported [20]. In previous reports, the DA with highest D3 affinity (pramipexole) appears to be more commonly implicated in pathological behaviors both in PD and in restless legs syndrome [23], but a large cross-sectional study found no difference between current use and risk for pathological behaviors between DAs [20]. Again, prior DA exposures and reasons for discontinuation were not reported.

The relationship between deep brain stimulation (DBS) of the subthalamic nucleus (STN) and impulse control disorders is complex, and it is the focus of several review papers [24, 25]. In general, a reduction in dopaminergic medication is seen after STN DBS, and with reduction or elimination of dopamine agonist therapy ICDs such as pathological gambling and others can improve [26–29]. However, several studies have noted de novo ICDs after DBS [30–32]. Interestingly, models of STN function [33] suggest that the STN modulates decision thresholds in proportion to reinforcement and decision conflict. Patients with STN DBS showed typical conflict-induced slowing in “win-win” computerized decision-making tasks with their DBS off, but 10 minutes after turning the DBS on, they exhibited less slowing and increased impulsive decision making in these same tasks [34]. Dopamine dysregulation syndrome (DDS) is a compulsive overuse of dopaminergic therapy. Preexisting DDS may or may not improve after STN DBS. Lim et al. found DDS remained unimproved or worsened in 12/17 patients after DBS, although this was a mix of STN and globus pallidus interna (GPi) DBS cases [32]. In the remaining 5/17 patients, DDS improved or resolved.

Discontinuation of the DA or significant adjustment in dosage is the mainstay of treatment intervention and appears to be required to achieve full remission or significant reduction in behaviors [35]. Even still, some patients exhibit persistent pathological behaviors. A study examining psychosocial outcomes in patents having exhibited pathological gambling found persistent financial and marital stress as a consequence of these behaviorsalthoughfull or partial resolution of the behaviors in all subjects followed [36].

Some authors group DA-associated behavior changes as disorders of impulse control, but careful examination of the behavioral issues reported in the medical literature and by our patients suggests a more complex behavioral derangement than a general disorder of impulse control. Patients appear to demonstrate a circumscribed obsessive-compulsion for a particular behavior. Most commonly, patients exhibit one particular obsession, but even in cases where two or more obsessions manifest, the more widespread injudicious decision making and excessive spontaneity that characterize a general disorder of impulse control are absent [20–23, 37, 38]. It may be that the neural systems mediating these pathologic behaviors are more closely aligned with punding (an intense fascination with meaningless movements or activities such as collecting, arranging, or taking apart objects), and one study suggested a strong relationship between punding and the expression of dyskinesias. Some studies suggest a D3 receptor-dependent response to L-dopa and dyskinesia, at least in monkeys [13].

Several recent studies have documented the importance of the brain circuits involved in reward and risky decision making, including, thalamic, striatal, and ventromedial frontal regions. Using fMRI, Reuter and colleagues compared pathological gamblers and control subjects and found that activation in regions such as the ventral striatum is inversely related to their pathological gambling severity, as if risks and rewards were less salient to pathological gamblers except at high enough magnitudes [39]. Another fMRI study had subjects play a game in which they decided to keep pumping up a virtual balloon or quit and collect reward points, with larger rewards associated with larger balloons [40]. Increased activation levels in insular, thalamic, striatal, and dorsolateral prefrontal regions bilaterally and medial prefrontal cortex/anterior cingulate regions correlated with increases in active risk taking. Functional imaging studies in PD patients have implicated similar brain regions [41, 42].

Voon et al. [38] studied PD patients with and without impulse control disorders (ICD) in a risk task involving a certain (e.g., +$100) or an uncertain outcome (e.g., 50/50 chance of winning either $200 or winning $0) for both gains (+$) and loss (−$) domains. PD patients without impulse control disorders behaved more similarly to healthy controls while they were on DA medications, making substantially more risky choices when they were confronted with losses than with gains, thereby showing “loss aversion” [43]. These same patients made highly similar choices in the gain versus the loss domains without loss aversion when they were off DA medications. PD patients with ICD showed more risk taking in the gain domain whether on or off medication, a pattern that was opposite to those of the healthy controls and PD patients without ICD. Moreover, PD patients with ICD also showed higher sensitivity to risk when they were on DA medications, displaying a steeper drop in the number of risky choices as the value at stake became higher and higher. In another study [44], PD patients without ICD were given the Iowa Gambling Task (IGT) while they were on or off medications. In this task, subjects chose between four decks of cards with various risk reward payoffs (i.e., risk disadvantageous (RD) decks with larger and frequent rewards but also infrequent large losses leading to long-term net losses, versus risk advantageous (RA) decks with smaller frequent rewards but also smaller infrequent losses leading to long-term net gains). PD patients off DA medications showed an appropriate decrease in choices for the risk-disadvantageous (RD) decks over trials. In contrast, PD patients on DA medications failed to show such outcome-contingent learning; instead, they kept on choosing the RD decks.

## 4. Implications for Other Disorders

Analogous behavioral changes arise spontaneously in the general population, where they are often termed “obsessive-compulsive disorder” or “addiction.” Obsessive-compulsive behaviors emerge in 30–50% of patients with Tourette syndrome [45], and recent PET imaging evidence suggests widespread dysregulation of extrastriatal dopamine response in subjects with Tourette syndrome relative to the response in control subjects [46]. As discussed above, this suggests a relationship between dysregulation of dopaminergic tone and obsessive-compulsive behavioral manifestations.

The mainstays of pharmacologic treatment for obsessive-compulsive disorder are antidepressant medications whose primary pharmacologic target is thought to be serotonin (5HT), a strategy that meets with varying success. Consideration of the interaction between 5HT and dopamine in the limbic system provides another perspective on how these medications may be mediating that success. Rodent studies implicate D2 and D3 receptor activity in models of obsessive-compulsive behavior and found that D2/3 agonism ameliorated these behavioral models [47, 48]. The emergence of similar behavioral drug-induced compulsive behaviors in PD patients with no history of such behaviors and that the prevalence of these behaviors appears to show a dose-dependent response adds further credence to the relevance of dopaminergic stimulation in idiopathic obsessive-compulsive behaviors. In addition to inhibiting reuptake of 5HT and norepinephrine, clomipramine acts as an antagonist at the D2 and D3 receptors, which may explain in part the efficacy of clomipramine in treating obsessive-compulsive disorder. Taken together, these observations suggest that modulation of specific dopaminergic receptors may hold promise for new medications directed against obsessive-compulsive behaviors.

Substance abuse literature suggests that liability to this disorder exists in 9–12% of humans. The D3 receptor does not appear to have a direct role in reinforcing the effects of drugs of abuse, but the role of the D3 receptor may be in processing novelty and in the environmental conditioning and associations that reinforce drugs of abuse, particularly those with psychostimulant effects. Initial studies in squirrel monkeys [49] and in rats [50] suggest an important role of the D3 and the closely related D2 receptor in mediating drug-related discriminatory behaviors, but they provide no evidence of a role of these receptors in direct reinforcement. The studies also suggest a role for these receptors in reinstatement of drug-use behaviors in abstinent animals. Taken together, these data suggest a potential role for D2/D3 specific ligands in decreasing relapse rates in abstinent drug abusers.

## 5. Conclusion

In the brief time since DAs have been widely used for treatment of PD, an important association between higher doses of these medications and the emergence of pathologic behaviors has been recognized. As outlined above, the D3 specificity of these medications and over-representation of the D3 receptor [51, 52] likely account for both the lower incidence of dyskinesias and also for the emergence of these pathological behaviors. This observation has important consequences for the safe use and monitoring of PD patients taking DA-agonists. Although the anatomical underpinning of this neural connectivity is incompletely understood, this observation also suggests potential therapeutic targets for obsessive-compulsive disorder and possibly for substance-based addictions. Advances in understanding the roles of specific dopamine receptors may also help to guide drug design for future atypical neuroleptics that aim to reduce side effects while improving efficacy.
